# Fluoroquinolone and beta-lactam antimicrobials induce different transcriptome profiles in *Salmonella enterica* persister cells

**DOI:** 10.1038/s41598-023-46142-8

**Published:** 2023-10-31

**Authors:** S. P. Mattiello, V. C. Barth, J. Scaria, C. A. S. Ferreira, S. D. Oliveira

**Affiliations:** 1 Laboratório de Imunologia e Microbiologia, Escola de Ciências da Saúde e da Vida, Pontifícia Universidade Católica do Rio Grande do Sul, PUCRS, Av. Ipiranga, 6681, Porto Alegre, 90619-900 Brazil; 2 Programa de Pós-Graduação em Biologia Celular e Molecular, Escola de Ciências da Saúde e da Vida, Pontifícia Universidade Católica do Rio Grande do Sul, PUCRS, Porto Alegre, Brazil; 3 College of Mathematics and Science, The University of Tennessee Southern, UTS, Pulaski, TN USA; 4 Department of Veterinary and Biomedical Sciences, South Dakota State University, SDSU, Brookings, SD USA; 5 Laboratório de Imunoterapia, Universidade Federal de Ciências da Saúde de Porto Alegre (UFCSPA), Porto Alegre, RS Brazil; 6 Department of Veterinary Pathobiology, Oklahoma State University, Stillwater, OK USA

**Keywords:** Biofilms, Cellular microbiology, Antimicrobials

## Abstract

Here, we investigate the transcriptome profiles of two *S*. Enteritidis and one *S.* Schwarzengrund isolates that present different persister levels when exposed to ciprofloxacin or ceftazidime. It was possible to note a distinct transcript profile among isolates, time of exposure, and treatment. We could not find a commonly expressed transcript profile that plays a role in persister formation after *S. enterica* exposure to beta-lactam or fluoroquinolone, as only three DEGs presented the same behavior under the conditions and isolates tested. It appears that the formation of persisters in *S. enterica* after exposure to ciprofloxacin is linked to the overexpression of genes involved in the SOS response (*recA*), cell division inhibitor (*sulA*), iron-sulfur metabolism (*hscA* and *iscS*), and type I TA system (*tisB*). On the other hand, most genes differentially expressed in *S. enterica* after exposure to ceftazidime appeared to be downregulated and were part of the flagellar assembly apparatus, citrate cycle (TCA cycle), glycolysis/gluconeogenesis, carbon metabolism, bacterial secretion system, quorum sensing, pyruvate metabolism pathway, and biosynthesis of secondary metabolites. The different transcriptome profiles found in *S. enterica* persisters induced by ciprofloxacin and ceftazidime suggest that these cells modulate their response differently according to each stress.

## Introduction

*Salmonella enterica* is one of the most common foodborne pathogens that causes self-limiting gastroenteritis and, in some cases, severe systemic infections. Based on 2014 data, the Centers for Disease Control and Prevention (CDC) estimates that nontyphoidal *Salmonella* causes approximately 1.35 million infections, resulting in 26,500 hospitalizations and 420 deaths annually in the United States^1^. Moreover, multi-resistant foodborne bacteria are increasing globally, partly as a result of antibiotic overuse, limiting therapeutic options and resulting in treatment failures^2^. In addition, a hard-to-treat bacterial infection that does not harbor antibiotic-resistance genes and often relapses even after several rounds of antibiotic treatment has been reported^3^. The recurrence of these infections may be related to the presence of persister cells^4–6^.

Persisters constitute a small subpopulation of cells that arises from a genetically identical bacterial culture and adopt a non-genetic and non-heritable bet-hedging strategy phenotype, surviving high doses of antibiotics by entering a transient and reversible slow or non-growth state^6,7^. This phenotype can be evidenced by a biphasic killing curve, where non-persisters are eliminated after the addition of lethal doses of a bactericidal antibiotic, while a persister subpopulation survives and resumes growth as the stressor is removed without undergoing any resistance-determining genetic change, unlike drug-resistant cells^8,9^. Persisters can be stochastically formed or triggered by multiple stress-induced factors, such as exposure to antimicrobial agents, nutritional deprivation (stringent response—SR), and changes in oxygen availability, pH, or carbon sources^10–13^.

It is widely acknowledged that the non-growth state found at least in part of these cells implies the modulation of transcriptional activity, including the shutting down of important pathways, especially those related to cell growth and energy production (ATP)^8,14–18^. Typically, low energy levels drive the reduction of translation, transcription, and DNA replication, leading to the inactivation of important antimicrobial targets that may contribute to persister formation^16,17^. Although persister cells have been described as metabolically inactive cells, different studies have demonstrated that they may, in fact, remain metabolically active^13,19,20^, and a dormancy state is not sufficient to explain the persistence phenomenon. The activation of efflux pumps^21^, quorum sensing cell signaling^22–25^, DNA repair (SOS response)^26–28^ and, toxin-antitoxin (TA) systems^13,27,29–35^ are believed to be involved in persister formation and/or regulation, and they can act alone or overlapped^36^.

The current knowledge about persistence is very fragmented, and important issues regarding the molecular mechanisms behind persister formation remain unexplored. Although controversial^14,37^, it is believed that the type II TA system is the main component involved in persister formation in *S*. *enterica*^30^. The TA systems *vapBC*, *relBE*^35^, and *shpAB* (*Salmonella* high persistence)^38^ have been described in *S.* Typhimurium persisters after ampicillin exposure. High expression of *relBE* and *shpAB* is usually activated by SR due to intracellular accumulation of a second messenger, (p)ppGpp, through the synthases RelA/SpoT, which activate the Lon protease. Lon degrades antitoxins, thereby releasing the associated toxins and inducing *S*. Typhimurium into a transient non- or slow growth inside vacuolar macrophages^13^. Recently, Rycroft et al. demonstrated the action of three acetyltransferase toxins (TacT, TacT2, and TacT3) on *S*. Typhimurium and *S*. Enteritidis, causing the acetylation of aminoacyl-tRNA molecules, which led to the inhibition of translation and persistence^34^.

Another important TA system involving the regulation of the SOS response, described in *Escherichia coli*, is the type I TA system TisB/IstR^27,32,33^. Fluoroquinolones kill bacteria by acting on DNA replication and causing damage that triggers the SOS response. This effect is mediated by RecA activation, which induces the autoproteolysis of the LexA repressor, stimulating the expression of proteins involved in repair, such as SulA, DinG, UvrABCD, RecABCD, and RuvABC^26,39,40^. In this case, TisB toxin is activated and forms channels in the cell membrane, causing an imbalance in the proton-motive force and decreasing cellular ATP levels, ultimately promoting the loss of antimicrobial activity^16,41^. Another important mechanism related to persister formation, particularly in *Staphylococcus aureus*, is the inactivation of tricarboxylic acid (TCA) cycle genes, supporting the hypothesis that depletion of ATP levels significantly increases the number of persister cells^42^.

Understanding the physiology of persisters is crucial to prevent their formation and recalcitrance to chronic infections. In this study, we investigated the transcriptome profiles of three isolates of *S. enterica* (two belonging to serovar *S*. Enteritidis and one to serovar *S.* Schwarzengrund), which demonstrated varying levels of persistence when exposed to ciprofloxacin or ceftazidime.

## Experimental procedures

### Bacterial isolates

*Salmonella enterica* serovar Enteritidis from porcine feces (4SA), chicken carcass (192), and *S.* Schwarzengrund (S58) from poultry flesh and bone meal were selected according to previous persistence assays by exposure to ciprofloxacin or ceftazidime^43^. *S.* Schwarzengrund (S58) was obtained from the veterinary diagnostic laboratory MercoLab (Cascavel, Brazil). The isolates were stored at -80 °C in Luria–Bertani (LB) medium (Sigma-Aldrich, Saint Louis, USA) supplemented with 20% DMSO.

### Genomic DNA isolation and sequencing

Genomic DNA was extracted from 1 mL of overnight *S. enterica* cultures using Qiagen DNeasy kits (Qiagen, Valencia, CA, USA), following the manufacturer’s instructions. DNA quality was analyzed using NanoDrop One (Thermo Scientific, WI, USA), quantified using a Qubit 3.0 fluorometer (Thermo Fisher Scientific Inc., MA, USA), and stored at − 20 °C until further use. Whole-genome sequencing (WGS) was performed using an Illumina MiSeq using V2 chemistry with 2 × 250 paired-end sequencing (Illumina, Inc., CA, USA). The sequenced reads were assembled using the default parameters of CLC Genomics Workbench 12.0 (Qiagen Bioinformatics). The resulting assembled genome was annotated using Prokka (Prokaryotic Genome Annotation) software version 1.13.7 (Supplementary Table S1). A pairwise FastANI comparison was performed with the three genomes sequenced here and the *Salmonella enterica* reference genome ASM694v2/ATCC 700720 (https://www.ncbi.nlm.nih.gov/datasets/genome/GCF_000006945.2/) (Supplementary data).

### Persister assay and RNA extraction

**Figure 1:**
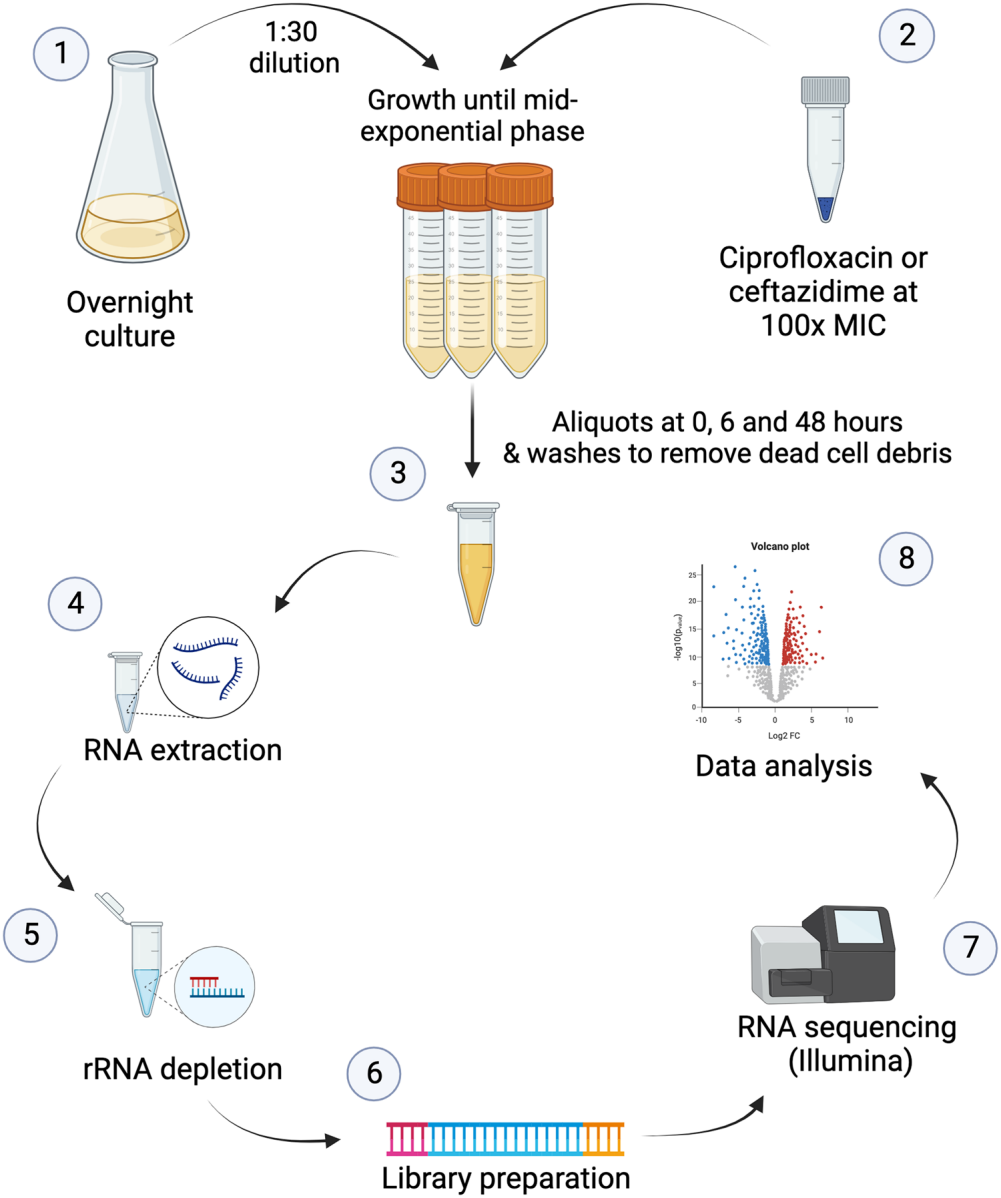
Experimental procedure for whole-transcriptome analysis of *Salmonella enterica* persisters induced by exposure to 100-fold MIC of ciprofloxacin or ceftazidime. Three biological replicates were performed for each time point. Overnight cultures were diluted 1:30 in fresh LB and incubated until mid-exponential growth phase (1). Ciprofloxacin or ceftazidime was added to a final concentration 100-fold above the MIC, and the samples were incubated for 48 h (2). 5-mL aliquots were removed at 0, 6, and 48 h after exposure (3) and treated with Bacteria RNAprotect prior to RNA extraction (4). RNA was treated with DNase I, and rRNA was depleted using the RiboZero Magnetic Kit for Gram-negative bacteria (5) before dsDNA synthesis and library preparation (6). RNASeq was performed with Illumina MiSeq platform (7) and the differentially expressed genes were identified with CLC Genomics Workbench 12.0 (8).

*Salmonella enterica* cultures at the mid-exponential phase were exposed to a 100-fold MIC (Minimal Inhibitory Concentration) of ciprofloxacin or ceftazidime according to the protocol described by Drescher et al.^43^, (Fig. 1). Briefly, persister levels were evaluated in planktonic cultures that were grown overnight in LB broth flasks. cultures were diluted at 1:30 and incubated without shaking at 37 °C for 2 h 30 min until the mid-exponential growth phase (approximately 10^8^ CFU/mL). Before antibiotic exposure (0 h) and at two time points (6 and 48 h), 5 mL-aliquots were removed and immediately treated for 5 min with RNAprotect Bacteria Reagent (Qiagen, 76506) before RNA extraction. The cultures were centrifuged at 11,088 × *g* for 5 min at 4 °C, and the pellet was washed with phosphate-buffered saline solution prior to stain with LIVE/DEAD BacLight (Bacterial Viability Kit; Life Technologies, Carlsbad, CA, USA) to access dead and live cells in the cultures. Persister cells were lysed with lysozyme (0.5 mg/mL) (Thermo Scientific, 89833) and 10% sodium dodecyl sulfate for 10 min at room temperature, followed by 10 min of incubation at 65 °C with phenol:chloroform:isoamyl alcohol (25:24:1) (Sigma-Aldrich, 516726). The cells were immediately placed on ice for 5 min and centrifuged at 21693 g for 10 min at 4 °C. The aqueous phase was removed and transferred to a new tube, to which 400 µL of cold chloroform were added, mixed and centrifuged at 21,693 × *g* for 10 min at 4 °C. One-tenth of the total volume of 3 M sodium acetate and 1 mL of absolute ethanol was added to the aqueous phase. The samples were centrifuged at 21,693 × *g* for 10 min at 4 °C, the supernatant was removed, and the pellet was washed with 70% ethanol and dried at room temperature for 5 min. The pellet was eluted in 50 µL of RNase-free water, treated with DNase I according to the manufacturer’s protocol (Invitrogen, 18068015) to remove genomic DNA, and stored at  − 80 °C until further use. Total RNA concentration was measured using NanoDrop One (Thermo Scientific), and integrity was verified by observing intact 16S rRNA and 23S rRNA bands by formaldehyde agarose gel electrophoresis.

### RNA-seq library preparation and sequencing

mRNA enrichment was performed using 5 µg of total RNA with the RiboMinus Bacteria Module (Invitrogen, A47335), according to the manufacturer’s specifications. The ribosomal-depleted mRNA was purified with RNeasy Power Clean (Qiagen, 13997-50) and used as a template for generation of the first-strand cDNA using random hexamer-primed anchored-dT primer (dT23VN) (New England BioLabs, S1330S) and ProtoScript II Reverse Transcriptase (New England BioLabs, M0368L), followed by synthesis of the second-strand cDNA (dsDNA) using the NEBNext Ultra II Non-directional RNA second strand synthesis module (New England BioLabs, E6111L), according to the manufacturer’s protocol.

Samples were quantified using a Qubit 3.0 fluorometer (Thermo Fisher Scientific) and adjusted to 0.3 ng/µL. Whole transcriptome sequencing libraries were prepared using the Nextera XT DNA Library Prep Kit for the fragmentation and tagmentation of cDNA. A PCR with 12 cycles of amplification was conducted to enrich the adapter-ligated fragments. A cleanup step using AMPure XP beads was performed to remove short library fragments. Library normalization was carried out according to the Illumina MiSeq protocol for Bead Normalization. Samples were pooled at equal volumes and denatured before dsDNA sequencing was performed on an Illumina MiSeq platform using a V2 kit with 2 × 250 paired-end chemistry (Illumina, Inc.).

The datasets generated and analyzed during the current study are available in the National Center for Biotechnology Information (NCBI) Sequence Read Archive (SRA) repository, BioProject accession: PRJNA992450.

### RNA-seq data analysis

RNA-seq data were analyzed using the standard parameters of CLC Genomics Workbench 12.0 (Qiagen Bioinformatics) to identify the differentially expressed genes between time points (0, 6, and 48 h) after exposure to ciprofloxacin or ceftazidime. The paired-end reads were mapped against their own assembled genome using the following parameters: mismatch cost = 2, insertion cost = 3, deletion cost = 3, length fraction = 0.8, similarity fraction = 0.8, strand specific = both, and maximum number of hits for a read = 10. Library sizes were normalized and expression values were measured in Reads Per Kilobase per million. Differentially expressed genes (DEGs) were identified by comparing sample transcriptomes before and after treatment. Statistically significant differences for DEGs were estimated using a cutoff for false discovery rate (FDR) *p*-value < 0.01 and an absolute value of log_2_ fold change > 1 or <  − 1. Data analysis was performed using custom codes written in R or Python 3 (Supplementary Table S2).

## Results

### Global gene expression profiles in *Salmonella enterica* persister cells vary according to the isolate

**Figure 2:**
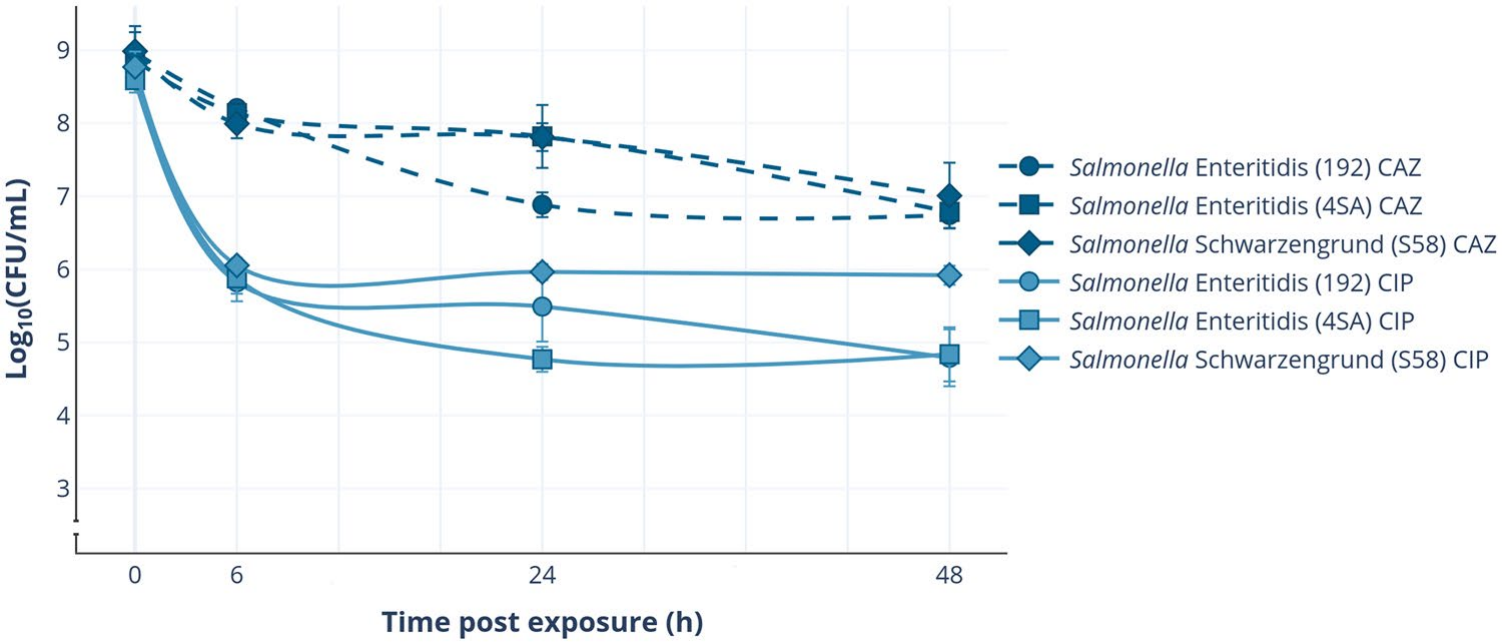
Effect of ciprofloxacin (CIP) or ceftazidime (CAZ) exposure on persister cell levels. Mid-log phase cultures of *Salmonella enterica* (time 0-h) were exposed to a 100-fold MIC of CIP or CAZ for 48 h. At each time point (6, 24, and 48 h), aliquots were collected to determine the fraction of surviving cells. The plotted values represent the mean of three biological replicates, and the error bars denote the standard deviation (± SD).

As previously reported^43^, there is high heterogeneity in persister levels among *S. enterica* isolates when cultured under the same conditions and exposed to the same antimicrobial, especially with ciprofloxacin. With this in mind, we selected two *S.* Enteritidis (4SA and 192) and one *S.* Schwarzengrund (S58) isolates that showed different persister levels after a 48-h exposure to a 100-fold MIC of ciprofloxacin or ceftazidime (ranging from 0.0127 to 0.1415% and 0.5952–1.057%, respectively) (Fig. 2).

**Figure 3:**
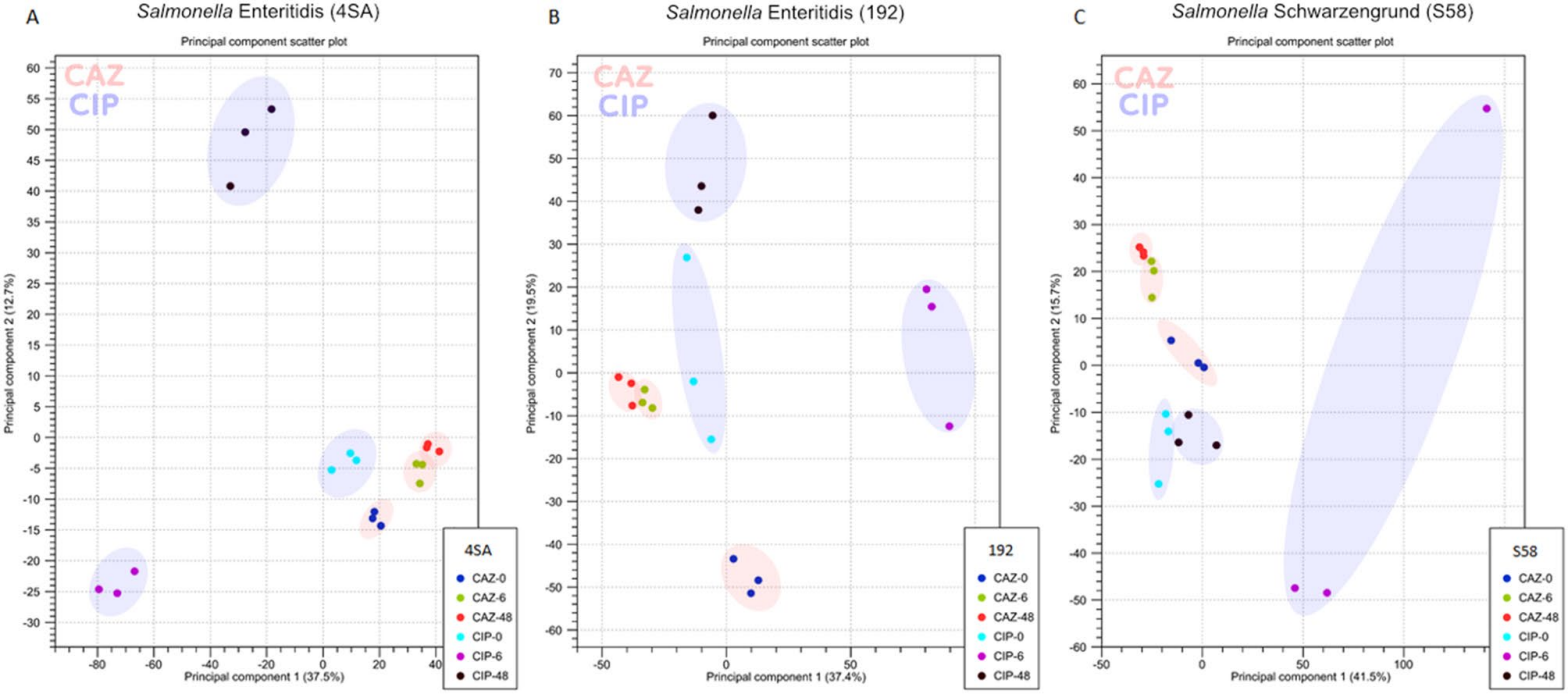
PCA plot depicting a comparison of persister transcriptome from (**A**) *Salmonella* Enteritidis (4SA), (**B**) *S.* Enteritidis (192), and (**C**) *S.* Schwarzengrund (S58) after exposure to 100-fold MIC of ceftazidime (CAZ) or ciprofloxacin (CIP) for 6 and 48 h, as well as before the addition of the antibiotic (0 h). The assay was performed in three biological replicates and analyzed using the CLC Genomics Workbench 12.0. The X-axis indicates principal component 1, and the Y-axis indicates principal component 2.

The transcriptome profiles of *S. enterica* persisters were analyzed after 6 and 48 h of exposure to 100-fold MIC of ciprofloxacin or ceftazidime, and compared with the samples before treatment. At these time points, only a small fraction of surviving cells remained in the sample after washing with phosphate-buffered saline (Fig. S1). The principal component analysis (PCA) plot was able to independently recapitulate the groups based on the similarities in their transcripts (Fig. 3A–C), summarizing the differences in transcription patterns of *S*. *enterica* persisters at three time points (0, 6, and 48 h) under different antibiotic treatments (Fig. 3). Remarkably, the expression profiles of persister cells exposed to ceftazidime tended to group more closely between the time points than those exposed to ciprofloxacin.

### Ceftazidime and ciprofloxacin treatment generate distinct isolate- or exposure-time associated transcription profiles

**Figure 4:**
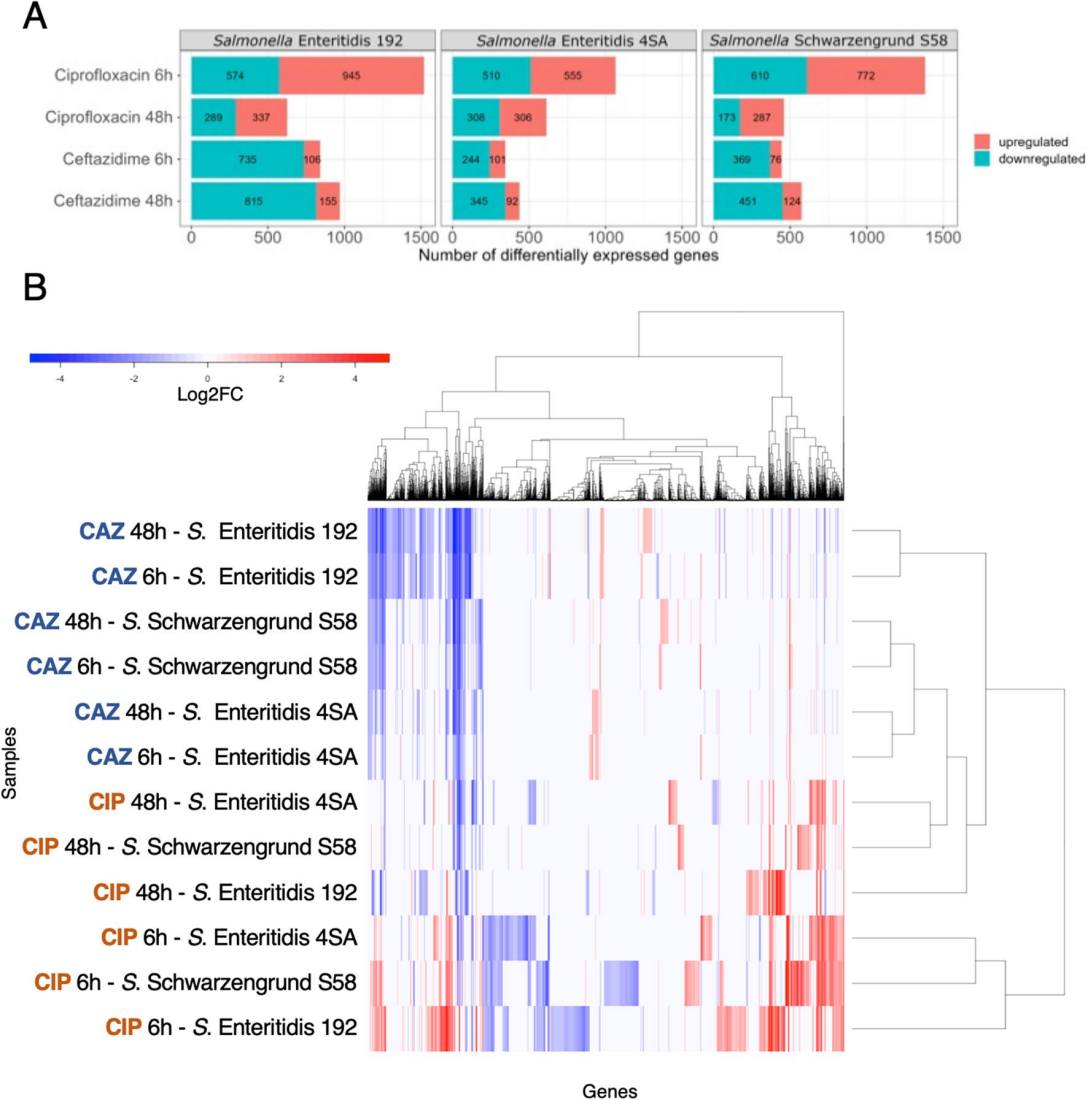
Transcriptomic analysis of *Salmonella enterica* persister cells exposed to ciprofloxacin (CIP) and ceftazidime (CAZ). (**A**) The Number of Differentially Expressed Genes (DEGs) in persister cells of *Salmonella enterica* (*S*. Enteritidis and *S*. Schwarzengrund) after 6 and 48 h of exposure to 100-fold MIC CIP or CAZ. Statistically significant expressed genes were filtered by a false discovery rate (FDR) *p*-value < 0.01 and an absolute value of log_2_ fold change > 1 or <  − 1. (**B**) Heatmap of DEGs. Hierarchical cluster analysis with Euclidean distance correlation and complete linkage. The heatmap displays the DEGs in *Salmonella enterica* persister cells after exposure to CAZ or CIP, statistically filtered by an FDR *p*-value ≤ 0.01, and an absolute value of log_2_ fold change > 1 or <  − 1. Different treatments and exposure times (rows) were clustered with the DEGs (columns). Colors indicate expression levels, ranging from highest (red) to lowest (blue). If the transcript was absent or not statistically significant in a given isolate, it is colored in white.

The PCA plots indicated a global change in the transcriptomic profiles of *S. enterica* cells under antimicrobial treatment. To understand the impact of each treatment and exposure time on persister formation, differential gene expression analysis was performed by filtering out non-significant variations using an FDR-adjusted *p*-value cut-off of < 0.01 and log_2_ fold change > 1 or <  − 1. A total of 3388 unique genes was differentially expressed (*p* < 0.01) among the persisters from all *S. enterica* isolates and time points (Supplementary Table S3). *S. enterica* persister cells in the ceftazidime-treated group presented a pronounced downregulation of transcripts across all isolates, which was not observed in the ciprofloxacin-treated group (Figs. 4A, S2). When comparing these DEGs from each group, the gene expression profiles from ciprofloxacin-treated persisters interestingly clustered in an isolate-independent manner, primarily grouping according to the time of antimicrobial exposure (Fig. 4B). Meanwhile, transcriptomic changes after ceftazidime exposure appeared to be more correlated within each isolate group.

### *Salmonella enterica* persister cells only presented four common transcripts after exposure to ciprofloxacin or ceftazidime

**Figure 5:**
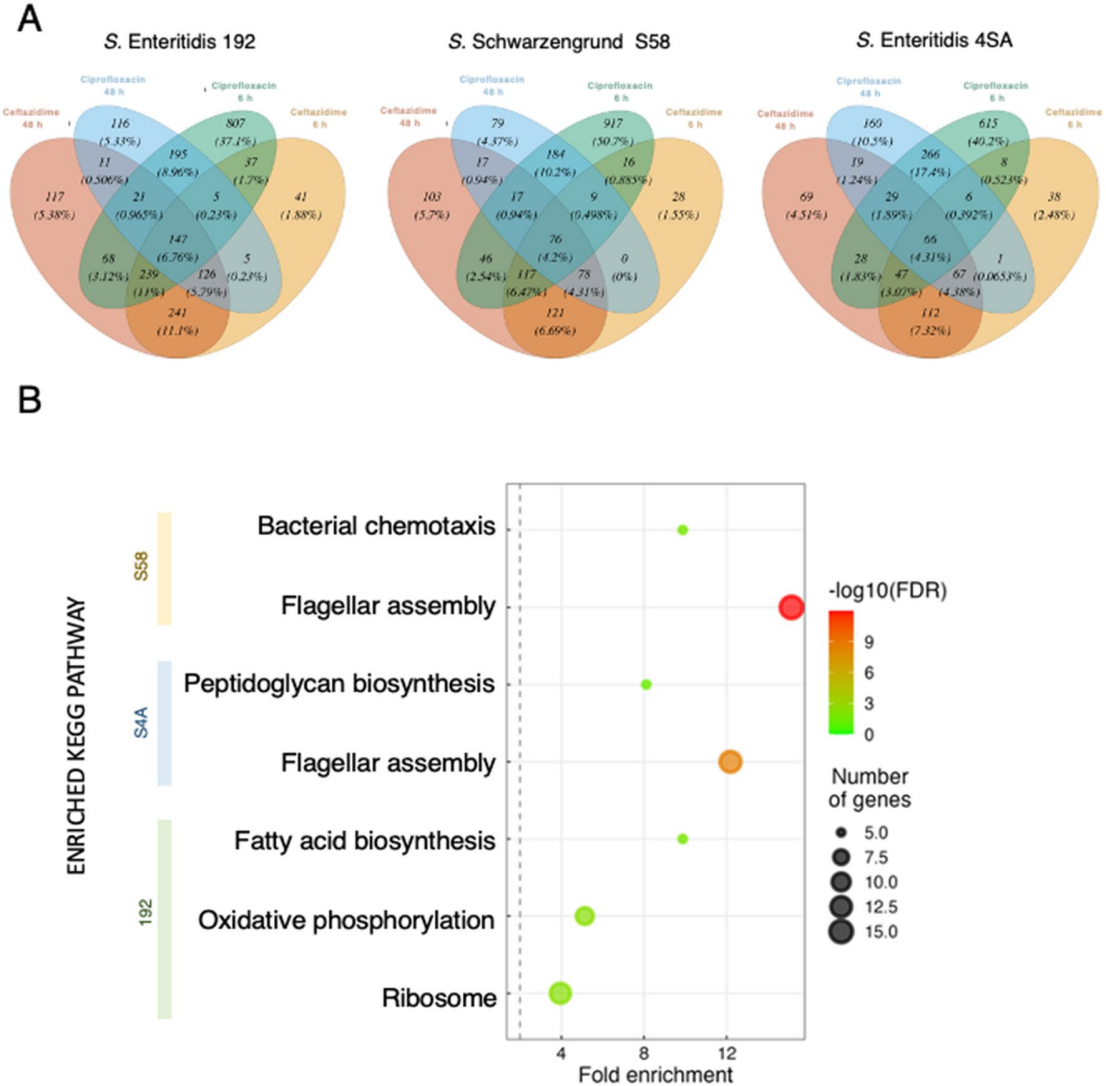
RNA-seq data analysis of *Salmonella enterica* persister cells. (**A**) Venn Diagram illustrating the overlap of the Differentially Expressed Genes (DEGs) identified in *Salmonella* Enteritidis (785-4SA), *S.* Enteritidis (182–192), and *S.* Schwarzengrund (796-S58) persister cells after exposure to 100-fold MIC of ciprofloxacin (CIP) or ceftazidime (CAZ) for 6 h and 48 h. DEGs were statistically filtered by a false discovery rate (FDR) *p*-value ≤ 0.01 and an absolute value of log_2_ fold change > 1 or <  − 1. (**B**) KEGG Pathway Enrichment Analysis performed using the DEGs that were common to all time-antibiotic treatments within each isolate group (*S*. Schwarzengrund S58, *S*. Enteritidis S4SA, *S*. Enteritidis 192). The size of the circles represents the number of genes in each statistically significant pathway, and the color indicates the FDR.

In order to identify a potential set of candidate genes universally associated with persistence, we compared DEGs in the persister cell fraction after each treatment. Across all treatments and time points, we found 66 common DEGs in *S*. Enteritidis (4SA), 147 in *S*. Enteritidis (192), and 76 in *S*. Schwarzengrund (S58) persisters (Fig. 5A). The vast majority of these DEGs were downregulated, as expected, due to the marked downregulation in the ceftazidime-treated persister cells. Pathway enrichment analysis revealed that these genes are mainly involved in flagellar assembly, bacterial chemotaxis, ribosome structure, oxidative phosphorylation, fatty acid metabolism, and peptidoglycan biosynthesis (Fig. 5B).

Interestingly, only four DEGs (*murG*, *focA*, *pspA*, and *nuoH*) were common to all *S. enterica* isolates after treatment with either ciprofloxacin or ceftazidime. Notably, both *murG* and *nuoH*, which encode a peptidoglycan biosynthesis enzyme and a subunit of NADH dehydrogenase I, respectively, were downregulated in all scenarios. In contrast, *pspA*, a phage shock stress regulator that senses cell membrane stress and has been reported to be important for persistence in *E. coli*^44^, was upregulated in all conditions. Also, previously described to influence persistence in *E. coli*^45^, *focA*, which encodes a formate transporter present in the inner membrane, exhibited a discrepant behavior. It showed both down- and up-regulation depending on the isolate and time of exposure to ciprofloxacin. However, under ceftazidime treatment, it showed upregulation in all conditions and isolates. Therefore, any effect derived from *foc*A expression involved in persister development or maintenance may not follow a single mechanism (Supplementary Table S5).

### *Salmonella* persister cells present antibiotic-specific transcription profiles

**Figure 6:**
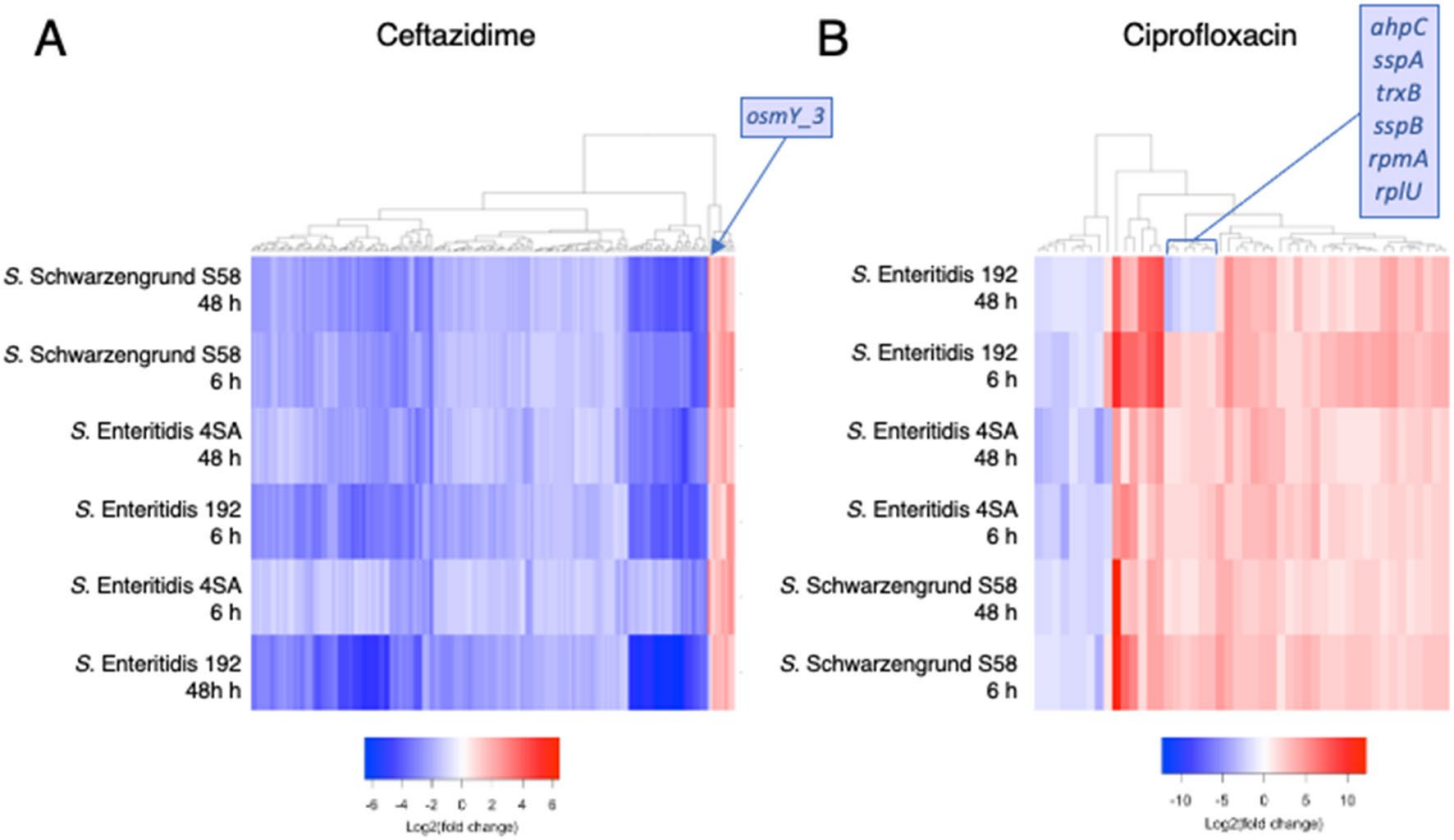
Heatmap showing ceftazidime- or ciprofloxacin-shared DEGs, that is, those found in all samples treated with either ceftazidime or ciprofloxacin. The heatmap location of genes that showed divergent expression exclusively in the *S*. Enteritidis 192 isolate (ie. *osmY_3*, *ahpC*, *sspA*, *trxB*, *sspB*, *rpmA*, and *rplU*) are indicated.

Considering the sharp separation between the persisters surviving ceftazidime and ciprofloxacin treatments, we sought to identify the signature response associated with each antibiotic by separately analyzing the shared DEGs within the two antibiotic groups. We observed a considerable overlap of DEGs when comparing the treatment groups (Fig. 5A). A total of 178 transcripts were found to be differentially regulated and common to all isolates of *S. enterica* persister cells after exposure to a 100-fold MIC of ceftazidime (Supplementary Table S4, Fig. 6A). The response in these core transcripts was strikingly similar, despite the differences in strains and time points, and they were predominantly downregulated. These DEGs are mainly involved in ribosome production, flagellar assembly, peptidoglycan biosynthesis, ATP synthesis, and secretion. Nearly all DEGs presented a common expression pattern across all isolates, except for one, *osmY_3*, in *S*. Enteritidis 192 (Fig. 6A). *osmY_3* encodes an osmotically inducible periplasmic protein of unknown function, and it was curiously upregulated exclusively in *S*. Enteritidis 192, contradicting gene expression in the other two strains. Moreover, a few genes were universally upregulated among all ceftazidime time points and strains: *otsB*, *pspA*, *dps*, *pspD*, *otsA*, *osmB*, *osmE*, *osmX*, and *spy*. The activation of these genes signals an ongoing osmotic stress, given that they are all involved in osmotic responses (*osmB*, *osmE*, and *osmX*), maintenance of cell envelope integrity (*pspA* and *pspD*), and trehalose biosynthesis (*ostA* and *ostB*), a disaccharide that also plays a role in controlling osmotic stress^46^.

Likewise, strikingly similar expression profiles were also observed in persister cells that survived ciprofloxacin treatment, although with fewer common DEGs (48) (Fig. 6B). However, the vast majority was upregulated in all samples, including transcripts that play pivotal roles in important processes, such as the SOS response (*recA* and *lexA*), TA systems (the toxin gene *tisB*), cell division inhibitor (*sulA*), carrier proteins (*yebE* and *yebF*), and iron-sulfur cluster assembly (*iscA*_2, *iscR*, *iscS*, isc*U*, and *hscB*) (Supplementary Table S5). On the other hand, the *nuo* operon, responsible for producing part of the respiratory chain complex I components, showed consistent downregulation in all strains, alongside *flhE* (part of the flagellar regulon) and *arnD* (involved in lipopolysaccharide modification).

Despite the similarities, a distinctive set of genes (*ahpC*, *trxB*, *rpmA*, *rplU*, *sspA*, and *sspB*) was also exclusively downregulated in the *S*. Enteritidis 192 isolate at the 48-h time point (Fig. 6B). These genes are involved in redox balance (alkyl hydroperoxidase reductase C, *ahpC*, and thioredoxin reductase, *trxB*), ribosomal formation (ribosomal protein L27, *rpmA*, and ribosomal protein L21, *rplU*), as well as secretion (*sspA* and *sspB*).

## Discussion

Despite several genetic approaches, the transcriptome of persister cells remains an understudied topic. Here, we provided insights from a whole transcriptome experiment, revealing possible transcripts that may take part in the establishment or maintenance of the persistence state in multiple strain and antibiotic combinations. Our results indicated heterogeneous responses, especially between the antibiotic treatments. This points to not one, but a diversity of gene expression profiles that vary according to the antibiotic treatment and/or genetic background, potentially contributing to the origin and maintenance of the persistence phenotype. In fact, we could only detect four genes that were differentially expressed in all strains and antibiotic treatments, and only three of them were fully concordant in their expression profiles across samples (*pspA* was upregulated, and *murG* and *nuoH* were downregulated in all treatments). This result reveals a lack of a single pathway responsible for multiple-drug persistence. Indeed, we were able to see similar profiles much more clearly within each class of drug, suggesting that the transcriptional changes that allow persister cell survival are, to a certain extent, antibiotic-specific. Consequently, eradication of persisters is likely better tackled by using a combination of antimicrobial drugs from distinct classes, as proposed in recent studies^47–49^.

Notwithstanding that only four DEGs were common to all conditions/isolates tested in this study, they bring some interesting clues upon persistence as a heterogeneous phenotype, as well as presenting a somewhat superposed physiological core. Downregulation of *murG* and *nuoH* may indicate that cell wall metabolism and the aerobic pulled proton motive force need to be altered^50,51^. Higher amounts of PspA also point to the need for membrane protection as a core survival strategy, as it was shown to form a protein carpet around damaged membranes, thus preventing proton leakage^52,53^. This activity seems to be conserved in PspA orthologous proteins and is suggested to be present even in chloroplasts^53^. The fourth universally common DEG found is *focA*, which participates in the import/export of formate from the cytoplasm to the periplasmic space, playing essential roles in fermentative metabolism and pH tunning^54^. Remarkably, or not so, *foc*A expression deviates from the other three common DEGs, as it did not show a common pattern of differential expression when the isolates were treated with ciprofloxacin. This indicates that not a single response and physiological network may be scaffolding persistence over time and/or when different genomes are challenged. It must also be highlighted that all four DEGs code for proteins present in the inner membrane-periplasmic space.

In our transcriptomic analysis, overall, we observed 3388 genes that were differentially expressed (log_2_ratio >  −/+ 1, FDR < 0.01) in the persistence state when compared to pre-treatment. This number is seemingly high, considering that the *Salmonella enterica* subsp. *enterica* reference genome typically contains ~ 4500 protein-coding genes. However, in face of the expected drastic differences in physiological states between the non-dividing persister group after antibiotic treatment and the rapidly multiplying pretreatment control group, the observed strong alterations in transcript profiles are plausible. Although we attribute most of these changes to the persistence phenotype, we cannot exclude other factors weighing on this transcriptome remodeling, such as density-dependent gene expression results (e.g., quorum sensing signaling). Moreover, accurately measuring transcript levels in persisters is a complex task because of the high background noise generated by antibiotic-induced cell death and debris. In our study, we attempted to minimize the impact of this noise by washing away most of the cell debris (as observed in Supplementary Fig. 1). We anticipate that this measure, along with the recent report that the half-life of *Salmonella* transcripts lies in the range of seconds to minutes^55^, would suffice to selectively capture persister cell transcripts. However, newer transcriptomic technologies (e.g., single-cell RNA sequencing) are required to profile persister cells more precisely in the future. These techniques, despite being highly expensive and technically challenging to perform in bacteria, would not only reduce the background from dead cells but would also provide an understanding of possible genetic interactions at the single-cell level as well as evaluate bona fide individual cell-to-cell variations. Total (“bulk”) RNA-seq presents the limitation of averaging the transcript abundance of the surviving population of cells, losing the heterogeneity information displayed by each individual cell. Indeed, high single-cell phenotypic heterogeneity has been observed in persister cells of *E. coli* using microfluidic fluorescence microscopy^56^. Still, we were able to detect consistent transcriptomic profiles in the persister cells that were treated by ceftazidime or ciprofloxacin. For instance, many genes upregulated in the surviving fractions of *S. enterica* upon ceftazidime exposure, regardless of the isolate, were related to the osmotic response. A potential reason for this response is that these genes were upregulated to combat the stress generated by the inhibition of cell wall synthesis by beta-lactams^57^, such as ceftazidime, thereby granting persister survival. Alternatively, it has been shown that *Salmonella* experiences high osmolarity stress inside macrophages^58^, a condition which curiously induces incredibly high levels (up to 1000-fold increase) of persister cells^13^. Therefore, it is also possible that the persistence phenotype in *Salmonella* is associated with the activation of a regulatory “survival” genetic network, which includes coping with osmotic pressure.

Conversely, ciprofloxacin-surviving persister cells showed strong signs of SOS response activation. For instance, *recA* and *lexA* genes were both upregulated in all ciprofloxacin-treated groups. These two genes are known to be inducible by ciprofloxacin treatment. We also observed elevated levels of transcripts that code for the TisB toxin (*tisB*) and the cell division inhibitor *sulA*, both of which are not only involved in growth arrest, but are also regulated by LexA^59^*.* TisB, for instance, has long been associated with increasing persister cell formation^33^ and accumulation of SulA has been reported as a key element to maintain the cell in a non-dividing state^40,60^. We also observed an interesting isolate-specific response involving the downregulation of six genes (*ahpC*, *trxB*, *rpmA*, *rplU*, *sspA*, and *sspB)* in *S*. Enteritidis 192. This isolate was also the only one that diverged in osmY_3 transcript levels upon ceftazidime treatment. This antagonistic response in one of the isolates implies that the genetic backgrounds of different isolates may contribute to different physiological interactions with genes from the core genome, generating heterogeneous and divergent responses, although converging to the same persistent phenotype.

In conclusion, this study provides new information about the heterogeneity of persister cells while also describing common profiles that are present and may sustain cells viable in a harsh environment. Individual genetic/epigenetic compositions, as well as the type and duration of antimicrobial stress, contribute differently to *Salmonella* persisters metabolism, although common mechanisms of adaptation seem to rely on the membrane and periplasm. Our study shed some light on the transcriptomic state of cells while in the presence of different antimicrobials. However, it also highlights the importance of investigating how diverse metabolic frameworks return to a common baseline phenotype once the stress is removed. Therefore, additional research is needed to uncover the specific transcriptional networks that are modulated, ultimately leading to cell resuscitation. Understanding the nature of persistence, especially its physiological strategies, may enable the development of efficient therapeutics to avoid recurrent infections, hopefully using the already available antimicrobials.

### Supplementary Information

Supplementary Legends.

Supplementary Figure S1.

Supplementary Figure S2.

Supplementary Table S1.

Supplementary Table S2.

Supplementary Table S3.

Supplementary Table S4.

Supplementary Table S5.

Supplementary Information.

## Data Availability

The sequencing datasets generated and analyzed during the current study conducted by Mattiello et al. is available in the National Center for Biotechnology Information (NCBI) Sequence Read Archive (SRA) repository, BioProject accession: PRJNA992450.

## References

[1] Collier SA (2021). Estimate of burden and direct healthcare cost of infectious waterborne disease in the United States. Emerg. Infect. Dis..

[2] Di Ciccio PA (2021). Antimicrobial-resistance of food-borne pathogens. Antibiotics (Basel).

[3] Grant SS, Hung DT (2013). Persistent bacterial infections, antibiotic tolerance, and the oxidative stress response. Virulence.

[4] Fauvart M, De Groote VN, Michiels J (2011). Role of persister cells in chronic infections: Clinical relevance and perspectives on anti-persister therapies. J. Med. Microbiol..

[5] Fisher RA, Gollan B, Helaine S (2017). Persistent bacterial infections and persister cells. Nat. Rev. Microbiol..

[6] Lewis K (2010). Persister cells. Annu. Rev. Microbiol..

[7] Brauner A, Fridman O, Gefen O, Balaban NQ (2016). Distinguishing between resistance, tolerance and persistence to antibiotic treatment. Nat. Rev. Microbiol..

[8] Lewis K (2012). Persister cells: Molecular mechanisms related to antibiotic tolerance. Handb. Exp. Pharmacol..

[9] Balaban NQ (2019). Definitions and guidelines for research on antibiotic persistence. Nat. Rev. Microbiol..

[10] Amato SM, Brynildsen MP (2014). Nutrient transitions are a source of persisters in *Escherichia coli* biofilms. PLoS One.

[11] Amato SM, Orman MA, Brynildsen MP (2013). Metabolic control of persister formation in *Escherichia coli*. Mol. Cell.

[12] Donamore BK, Gallo SW, Abreu Ferreira PM, Sanchez Ferreira CA, de Oliveira SD (2018). Levels of persisters influenced by aeration in *Acinetobacter calcoaceticus-baumannii*. Future Microbiol..

[13] Helaine S (2014). Internalization of *Salmonella* by macrophages induces formation of nonreplicating persisters. Science.

[14] Conlon BP (2016). Persister formation in *Staphylococcus aureus* is associated with ATP depletion. Nat. Microbiol..

[15] Liu X (2020). The potassium transporter KdpA affects persister formation by regulating ATP levels in *Mycobacterium marinum*. Emerg. Microbes Infect..

[16] Shan Y (2017). ATP-Dependent Persister Formation in *Escherichia coli*. mBio.

[17] Wilmaerts D, Windels EM, Verstraeten N, Michiels J (2019). General mechanisms leading to persister formation and awakening. Trends Genet..

[18] Manuse S (2021). Bacterial persisters are a stochastically formed subpopulation of low-energy cells. PLoS Biol..

[19] Manina G, Dhar N, McKinney JD (2015). Stress and host immunity amplify *Mycobacterium tuberculosis* phenotypic heterogeneity and induce nongrowing metabolically active forms. Cell Host Microbe.

[20] Stapels DAC (2018). *Salmonella* persisters undermine host immune defenses during antibiotic treatment. Science.

[21] Byrd BA (2021). The AcrAB-TolC efflux pump impacts persistence and resistance development in stationary-phase *Escherichia coli* following delafloxacin treatment. Antimicrob. Agents Chemother..

[22] Bhargava N, Sharma P, Capalash N (2014). Pyocyanin stimulates quorum sensing-mediated tolerance to oxidative stress and increases persister cell populations in *Acinetobacter baumannii*. Infect. Immun..

[23] Leung V, Levesque CM (2012). A stress-inducible quorum-sensing peptide mediates the formation of persister cells with noninherited multidrug tolerance. J. Bacteriol..

[24] Walawalkar YD, Vaidya Y, Nayak V (2016). Response of *Salmonella* Typhi to bile-generated oxidative stress: Implication of quorum sensing and persister cell populations. Pathog. Dis..

[25] Xu T (2017). The Agr quorum sensing system represses persister formation through regulation of phenol soluble modulins in *Staphylococcus aureus*. Front. Microbiol..

[26] Dorr T, Lewis K, Vulic M (2009). SOS response induces persistence to fluoroquinolones in *Escherichia coli*. PLoS Genet..

[27] Edelmann D, Oberpaul M, Schaberle TF, Berghoff BA (2021). Post-transcriptional deregulation of the *tisB/istR*-1 toxin-antitoxin system promotes SOS-independent persister formation in *Escherichia coli*. Environ. Microbiol. Rep..

[28] Podlesek Z, Zgur Bertok D (2020). The DNA damage inducible SOS response is a key player in the generation of bacterial persister cells and population wide tolerance. Front. Microbiol..

[29] Cho J, Carr AN, Whitworth L, Johnson B, Wilson KS (2017). MazEF toxin-antitoxin proteins alter *Escherichia coli* cell morphology and infrastructure during persister formation and regrowth. Microbiology.

[30] Cheverton AM (2016). A *Salmonella* toxin promotes persister formation through acetylation of tRNA. Mol. Cell.

[31] Gelens L, Hill L, Vandervelde A, Danckaert J, Loris R (2013). A general model for toxin-antitoxin module dynamics can explain persister cell formation in *E. coli*. PLoS Comput. Biol..

[32] Wagner EG, Unoson C (2012). The toxin-antitoxin system *tisB-istR1*: Expression, regulation, and biological role in persister phenotypes. RNA Biol..

[33] Dorr T, Vulic M, Lewis K (2010). Ciprofloxacin causes persister formation by inducing the TisB toxin in *Escherichia coli*. PLoS Biol..

[34] Rycroft JA (2018). Activity of acetyltransferase toxins involved in *Salmonella* persister formation during macrophage infection. Nat. Commun..

[35] Silva-Herzog E, McDonald EM, Crooks AL, Detweiler CS (2015). Physiologic stresses reveal a *Salmonella* persister state and TA family toxins modulate tolerance to these stresses. PLoS One.

[36] Van den Bergh B, Fauvart M, Michiels J (2017). Formation, physiology, ecology, evolution and clinical importance of bacterial persisters. FEMS Microbiol. Rev..

[37] Goormaghtigh F (2018). Reassessing the role of type II toxin-antitoxin systems in formation of *Escherichia coli* Type II persister cells. mBio.

[38] Slattery A, Victorsen AH, Brown A, Hillman K, Phillips GJ (2013). Isolation of highly persistent mutants of *Salmonella enterica* serovar typhimurium reveals a new toxin-antitoxin module. J. Bacteriol..

[39] Wilmaerts D, Focant C, Matthay P, Michiels J (2022). Transcription-coupled DNA repair underlies variation in persister awakening and the emergence of resistance. Cell Rep..

[40] Mohiuddin SG, Massahi A, Orman MA (2022). *lon* deletion impairs persister cell resuscitation in *Escherichia coli*. mBio.

[41] Wang M, Chan EWC, Wan Y, Wong MH, Chen S (2021). Active maintenance of proton motive force mediates starvation-induced bacterial antibiotic tolerance in *Escherichia coli*. Commun. Biol..

[42] Wang Y (2018). Inactivation of TCA cycle enhances *Staphylococcus aureus* persister cell formation in stationary phase. Sci. Rep..

[43] Drescher SPM, Gallo SW, Ferreira PMA, Ferreira CAS, Oliveira SD (2019). *Salmonella enterica* persister cells form unstable small colony variants after in vitro exposure to ciprofloxacin. Sci. Rep..

[44] Vega NM, Allison KR, Khalil AS, Collins JJ (2012). Signaling-mediated bacterial persister formation. Nat. Chem. Biol..

[45] Chowdhury N, Kwan BW, Wood TK (2016). Persistence increases in the absence of the alarmone guanosine tetraphosphate by reducing cell growth. Sci. Rep..

[46] Howells AM (2002). Role of trehalose biosynthesis in environmental survival and virulence of *Salmonella enterica* serovar typhimurium. Res. Microbiol..

[47] Baek MS, Chung ES, Jung DS, Ko KS (2020). Effect of colistin-based antibiotic combinations on the eradication of persister cells in *Pseudomonas aeruginosa*. J. Antimicrob. Chemother..

[48] Chung ES, Ko KS (2019). Eradication of persister cells of *Acinetobacter baumannii* through combination of colistin and amikacin antibiotics. J. Antimicrob. Chemother..

[49] Gallo SW, Ferreira CAS, de Oliveira SD (2017). Combination of polymyxin B and meropenem eradicates persister cells from *Acinetobacter baumannii* strains in exponential growth. J. Med. Microbiol..

[50] Sazanov LA (2015). A giant molecular proton pump: structure and mechanism of respiratory complex I. Nat Rev Mol Cell Biol.

[51] Zhang L (2021). An alternative and conserved cell wall enzyme that can substitute for the lipid II synthase MurG. mBio.

[52] Kobayashi R, Suzuki T, Yoshida M (2007). *Escherichia coli* phage-shock protein A (PspA) binds to membrane phospholipids and repairs proton leakage of the damaged membranes. Mol. Microbiol..

[53] Junglas B (2020). IM30 IDPs form a membrane-protective carpet upon super-complex disassembly. Commun. Biol..

[54] Kammel M, Sawers RG (2022). The FocA channel functions to maintain intracellular formate homeostasis during *Escherichia coli* fermentation. Microbiology.

[55] Jenniches L (2023). Improved RNA stability estimation through Bayesian modeling reveals most bacterial transcripts have sub-minute half-lives. bioRxiv.

[56] Goormaghtigh F, Van Melderen L (2019). Single-cell imaging and characterization of *Escherichia coli* persister cells to ofloxacin in exponential cultures. Sci. Adv..

[57] Wong F (2021). Understanding beta-lactam-induced lysis at the single-cell level. Front. Microbiol..

[58] Lee EJ, Choi J, Groisman EA (2014). Control of a *Salmonella* virulence operon by proline-charged tRNA(Pro). Proc. Natl. Acad. Sci. U. S. A..

[59] Vogel J, Argaman L, Wagner EG, Altuvia S (2004). The small RNA IstR inhibits synthesis of an SOS-induced toxic peptide. Curr. Biol..

[60] Schoemaker JM, Gayda RC, Markovitz A (1984). Regulation of cell division in *Escherichia coli*: SOS induction and cellular location of the *sul*A protein, a key to lon-associated filamentation and death. J. Bacteriol..

