# Supplementary material for: Fluoroquinolone and beta-lactam antimicrobials induce different transcriptome profiles in Salmonella enterica persister cells
Source: Sci Rep. 2023 Oct 31;13:18696. doi: 10.1038/s41598-023-46142-8 (PMC10618250; doi:10.1038/s41598-023-46142-8)
Supplement: Supplementary file 1 — Supplementary Legends. [file 41598_2023_46142_MOESM1_ESM.docx]

**Legends for supplementary figures and tables**

**Figure S1. The LIVE/DEAD Baclight assay was performed on an isolate of *Salmonella* Enteritidis 4SA.** The culture in the exponential phase (pre-antibiotic treatment) was either inactivated by ethanol as a control (ethanol inactivated) or treated with 100 times the MIC of ceftazidime or ciprofloxacin for 6 or 48 h. Intact cells are stained by SYTO9 (left panel, in green) and cells with compromised membranes are stained by propidium iodide (PI, right panel, in red), as an indicator of non-viable cells. Arrows indicate the remaining surviving (persister) cells after washing with phosphate-buffered saline.

**Figure S2. Volcano plots depicting transcriptomic changes in three isolates (*S*. Enteritidis S4A, *S*. Enteritidis 192, *S*. Schwarzengrund S58) after treatment with ceftazidime (CAZ) or ciprofloxacin (CIP)**. Antibiotic treatments lasted for 6 or 48 h to select surviving persister cells. Each circle represents a transcript detected using RNA sequencing. Statistically significant (at least a 2-fold difference compared to the control and a q-value < 0.01) are colored in red. Non-statistically significant transcripts are colored in blue (when the q-value threshold was met), or in green (when only the fold change threshold was met) or in gray (when neither threshold was met).

**Supplementary Table S1.** Statistical summary of Whole Genome Sequencing across different *Salmonella enterica* isolates.

**Supplementary Table S2.** Statistical summary of the RNA-Seq across different *Salmonella enterica* isolates.

**Supplementary Table S3.** RNA-seq results showing the expression of all detected genes at 0, 6, and 48 h. The S3 table shows an overview of all the transcripts detected in *Salmonella enterica* persister cells within mapped count reads in assembled RNA-Seq data.

**Supplementary Table S4**. Differentially expressed genes (DEG) in persister cells from distinct isolates of *Salmonella enterica* after exposure to ceftazidime (CAZ) in different time points. Log_2_ fold change > 1 or < -1 of the differentially expressed genes was statistically filtered by a false discovery rate *p*-value ≤ 0.01.

**Supplementary Table S5**. Differentially expressed genes (DEG) persister cells from distinct isolates of *Salmonella enterica* after exposure to ciprofloxacin (CIP) in different time points. Log_2_ fold change > 1 or < -1 of the differentially expressed genes was statistically filtered by a false discovery rate *p*-value ≤ 0.01.
