# Supplementary figures and images for: Fluoroquinolone and beta-lactam antimicrobials induce different transcriptome profiles in Salmonella enterica persister cells
Source: Sci Rep. 2023 Oct 31;13:18696. doi: 10.1038/s41598-023-46142-8 (PMC10618250; doi:10.1038/s41598-023-46142-8)

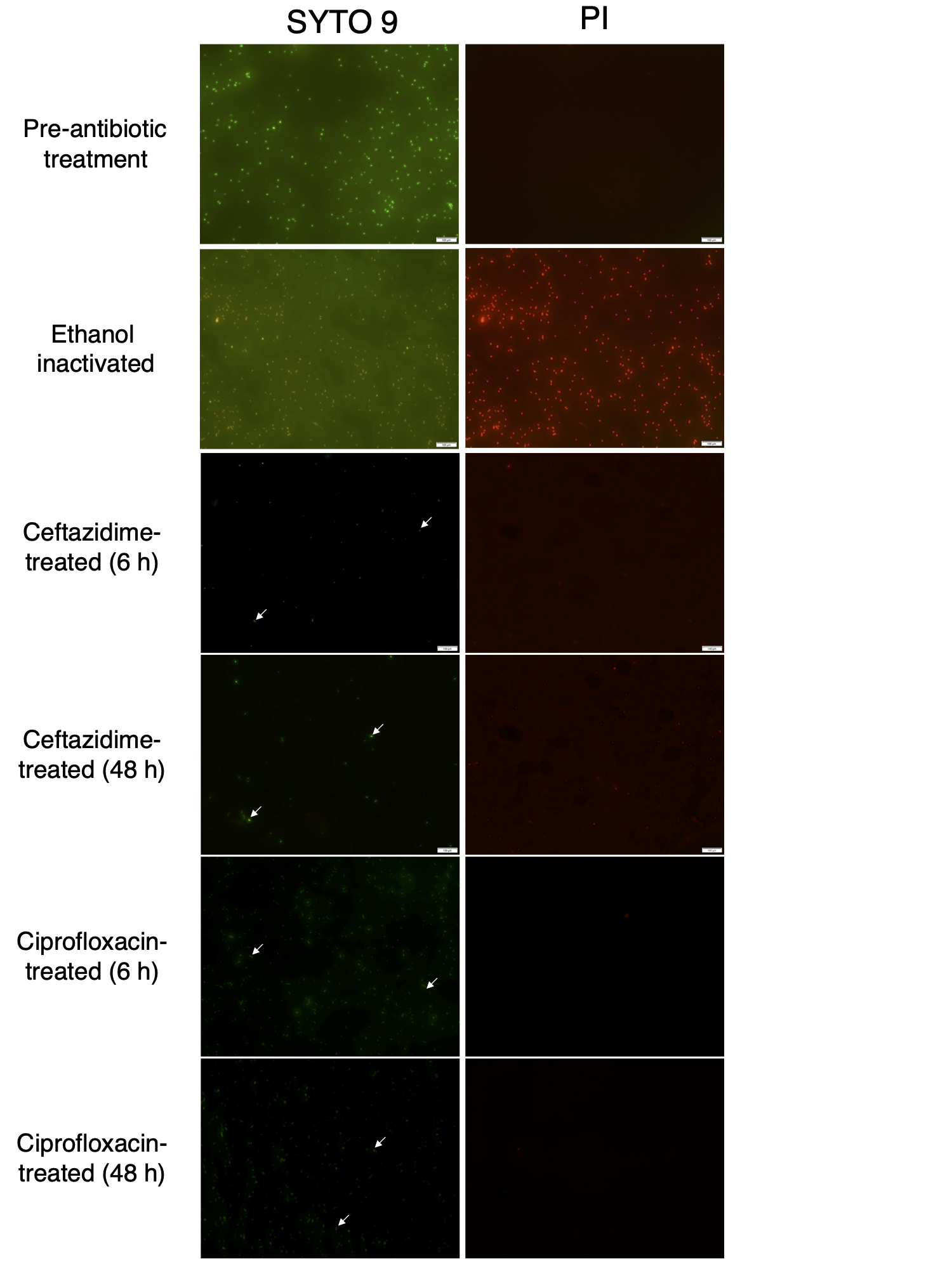

Supplement: Supplementary file 2 — Supplementary Figure S1. [file 41598_2023_46142_MOESM2_ESM.tiff]

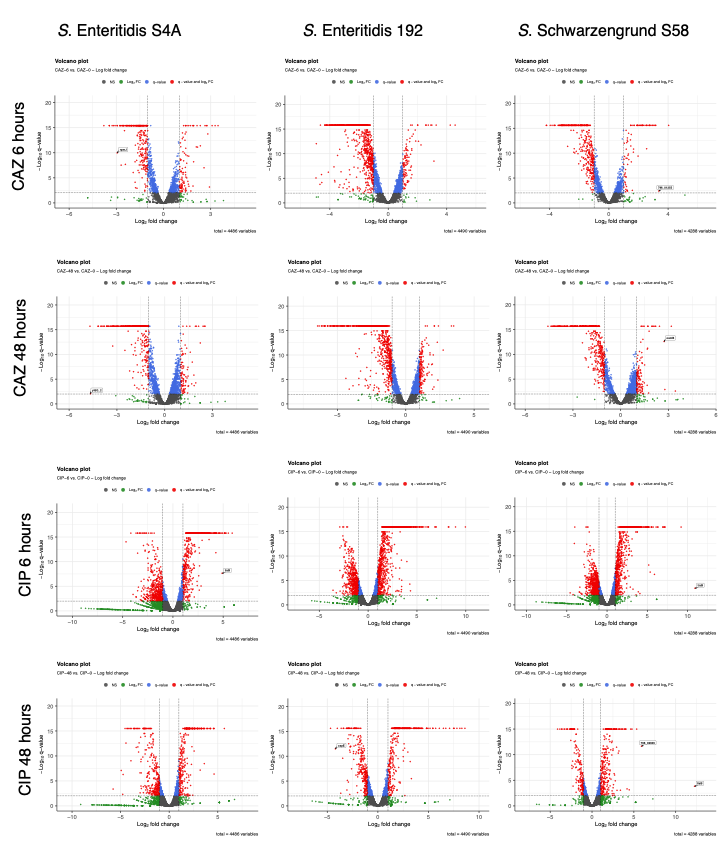

Supplement: Supplementary file 3 — Supplementary Figure S2. [file 41598_2023_46142_MOESM3_ESM.tiff]
