# Supplementary material for: Fluoroquinolone and beta-lactam antimicrobials induce different transcriptome profiles in Salmonella enterica persister cells
Source: Sci Rep. 2023 Oct 31;13:18696. doi: 10.1038/s41598-023-46142-8 (PMC10618250; doi:10.1038/s41598-023-46142-8)
Supplement: Supplementary file 9 — Supplementary Information. [file 41598_2023_46142_MOESM9_ESM.pdf]

Supplementary data

FastANI comparison of *Salmonella* strains with *Salmonella enterica* reference genome ASM694v2 / ATCC 700720 (https://www.ncbi.nlm.nih.gov/datasets/genome/GCF_000006945.2/)

QUERY	REFERENCE	ANI ESTIMATE	MATCHES	TOTAL
S. Schwarzengrund S58	GCF_000006945.2_ASM694v2	98.2649	1426	1516
S. Enteritidis 4SA	GCF_000006945.2_ASM694v2	98.8289	1475	1559
S. Enteritidis 192	GCF_000006945.2_ASM694v2	98.8467	1482	1548

S. Schwarzengrund S58 (top) vs. ASM694v2 (bottom)

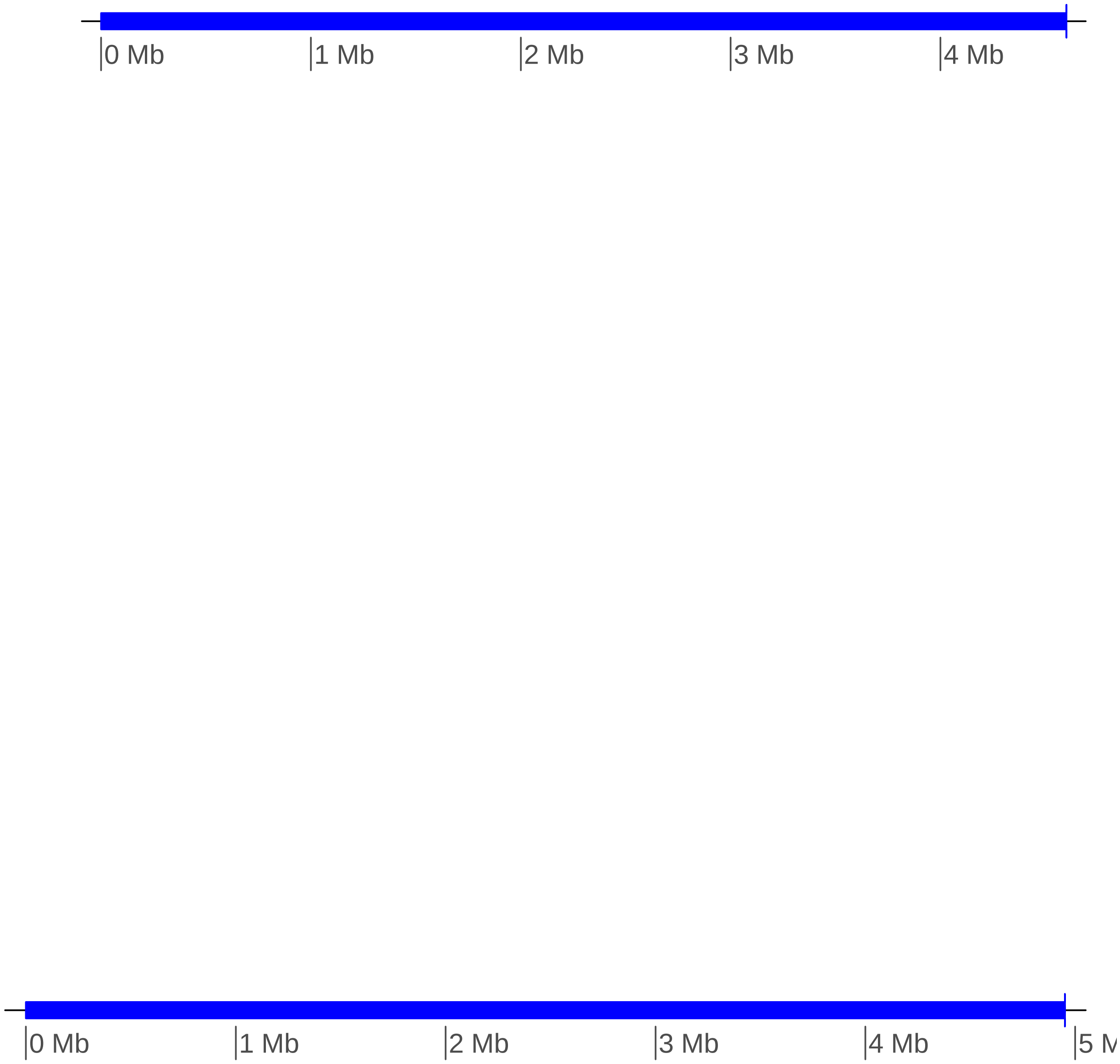

S. Enteritidis 4SA (top) vs. ASM694v2 (bottom)

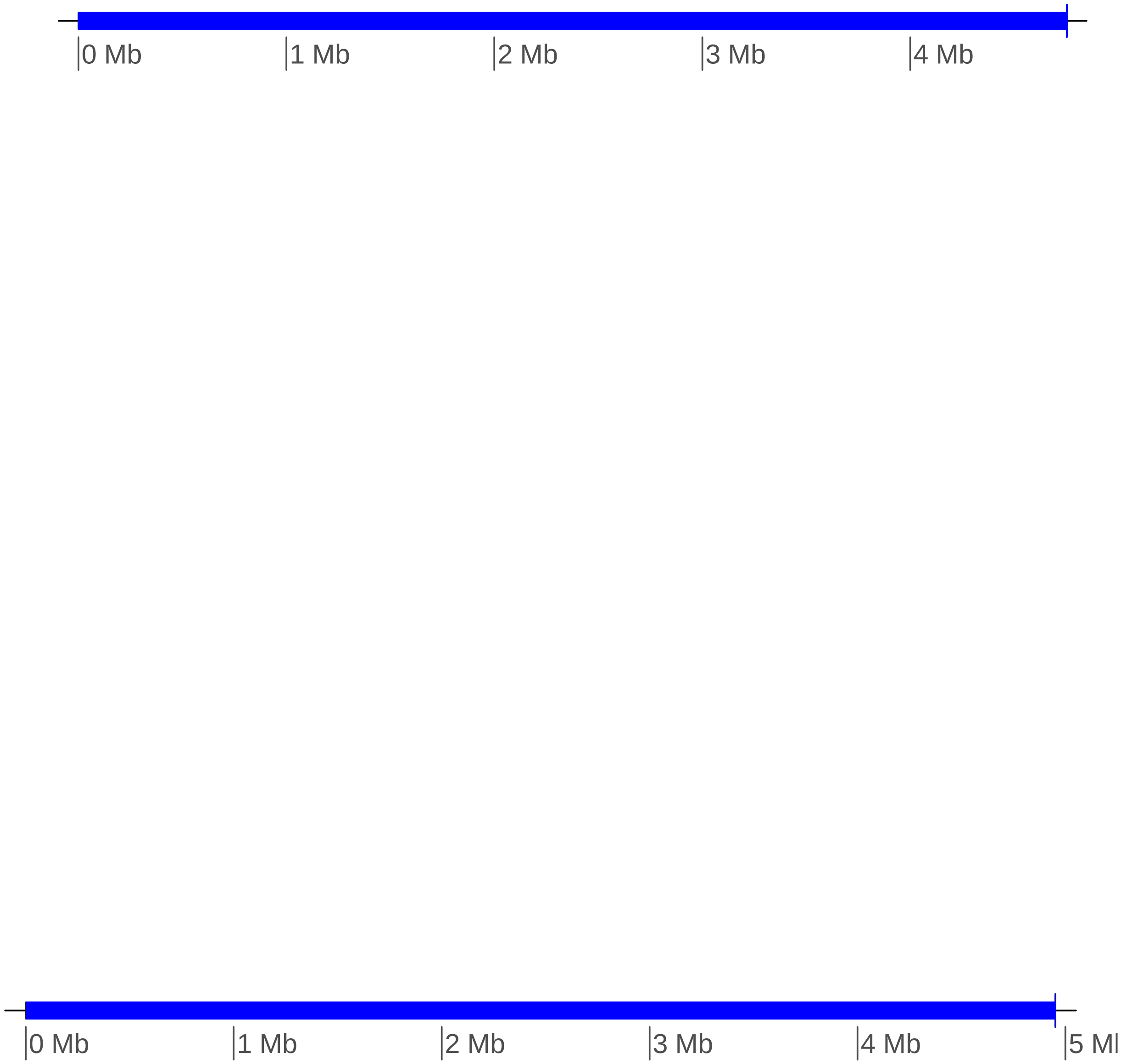

S. Enteritidis 192 (top) vs. ASM694v2 (bottom)

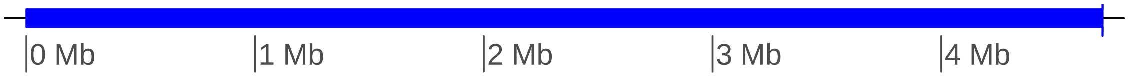
0 Mb
1 Mb
2 Mb
3 Mb
4 Mb

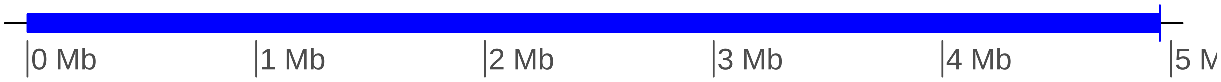
0 Mb
1 Mb
2 Mb
3 Mb
4 Mb
5 M
